# Insulin regulates multiple signaling pathways leading to monocyte/macrophage chemotaxis into the wound tissue

**DOI:** 10.1242/bio.026187

**Published:** 2017-11-03

**Authors:** Yan Liu, Sandeep Dhall, Anthony Castro, Alex Chan, Raquelle Alamat, Manuela Martins-Green

**Affiliations:** 1 Department of Burn and Plastic Surgery, ShangHai JiaoTong University School of Medicine Ruijin hospital, Shanghai, P.R.China 200025; 2 Department of Cell Biology and Neuroscience, University of California, Riverside, CA 92521, USA

**Keywords:** Inflammation, Wound healing, Neutrophils, GTPase, Rac1, Diabetes

## Abstract

Wound healing is a complex process that involves sequential phases that overlap in time and space and affect each other dynamically at the gene and protein levels. We previously showed that insulin accelerates wound healing by stimulating faster and regenerative healing. One of the processes that insulin stimulates is an increase in monocyte/macrophage chemotaxis. In this study, we performed experiments *in vivo* and *in vitro* to elucidate the signaling transduction pathways that are involved in insulin-induced monocyte/macrophage chemotaxis. We found that insulin stimulates THP-1 cell chemotaxis in a dose-dependent and insulin receptor-dependent manner. We also show that the kinases PI3K-Akt, SPAK/JNK, and p38 MAPK are key molecules in the insulin-induced signaling pathways that lead to chemoattraction of the THP-1 cell. Furthermore, both PI3K-Akt and SPAK/JNK signaling involve Rac1 activation, an important molecule in regulating cell motility. Indeed, topical application of Rac1 inhibitor at an early stage during the healing process caused delayed and impaired healing even in the presence of insulin. These results delineate cell and molecular mechanisms involved in insulin-induced chemotaxis of monocyte/macrophage, cells that are critical for proper healing.

## Abstract (precis)

**Summary:** Insulin regulates multiple signaling pathways leading to monocyte/macrophage chemotaxis into the wound tissue, involving -Akt, SPAK/JNK, and p38 MAPK which in turn are involved in Rac1 activation. Furthermore, these results augment our understanding of the insulin-regulated wound inflammatory response.

## INTRODUCTION

Wound healing involves a series of well-orchestrated cellular and molecular processes: hemostasis, inflammation, granulation tissue formation and angiogenesis, wound contraction and remodeling; which all occur in an orderly manner. At early stages of healing, inflammation plays an essential and integral role in initiating and regulating healing progression and determining how well a wound will heal. Although neutrophils, monocyte/macrophages, mast cells, and lymphocytes all actively participate in the inflammatory response, monocyte/macrophages play a critical and strong regulatory role. Monocytes, when attracted to the wound site, are activated to differentiate into macrophages. Molecules that attract and activate monocyte/macrophages include: inflammatory mediators, such as the chemokines monocyte chemoattractant protein one (MCP-1), macrophage inflammatory protein-1 (MIP)-1α, the growth factor macrophage colony stimulating factor (M-CSF); pathogens such as bacteria and fungi; and fragments from extracellular matrix (ECM) molecules such as collagen and fibronectin (Barrientos et al., 2008; Diegelmann and Evans, 2004; Suh et al., 2005).

Macrophages play a scavenger role during the early stages of wound healing and release various enzymatic proteins that propel healing to the next step. They also phagocytize and eliminate pathogenic organisms, tissue debris, apoptotic neutrophils, and other inflammatory cells. Furthermore, they are capable of controlling the inflammatory response in the wound by preventing excessive inflammation that can cause impaired healing. Chronic inflammation, induced by persistent monocyte infiltration, and macrophage accumulation are often associated with tissue destruction and fibrosis (Diegelmann and Evans, 2004). Monocytes/macrophages also regulate wound re-epithelialization and remodeling; therefore, acceleration of wound healing by manipulation of monocyte/macrophage function may be a good approach to improving healing (Suh et al., 2005; Hoare et al., 2016).

The effect of insulin on increasing the rate of wound healing has been observed in different animal models, including mice, rats, rabbits and horses, and in different wound types, such as diabetic and non-diabetic, burn wounds, excision wounds, fractures, and cutaneous ulcerations (Grewal et al., 1972; Hanam et al., 1983; Zhang et al., 2007; Liu et al., 2004; Paul, 1966; Lopez and Mena, 1968; Edmonds, 1976; Stunkle and Wray, 1965). The effectiveness of insulin treatment on accelerating wound healing has been confirmed on burn patients as shown by shorter donor site healing time (Pierre et al., 1998). Other studies, as well as our own previous work, show that insulin accelerates wound healing by regulating multiple cellular functions in multiple aspects of the healing process (Dhall et al., 2015; Liu et al., 2009a,b; Chen et al., 2012a,b). Indeed, we showed that burns treated with insulin-containing PLGA dressings every 3 days for 9 days have faster closure, decreased oxidative stress and the pattern of neutrophil inflammatory response suggests faster clearing of the burned dead tissue. We also observe faster resolution of the pro-inflammatory macrophages and found that insulin stimulates collagen deposition and maturation with basket weave-like organization (normal skin), rather than parallel alignment and cross-linking (scar tissue).

Insulin has been used extensively in humans. The safety, along with the low cost and potent regulation of wound healing processes, points to the promise that insulin can be used for the treatment of acute and problematic wounds. Since insulin modulates macrophage function, in this study we elucidate the signaling transduction pathways involved in insulin-induced monocyte chemotaxis. Monocytes circulate in the blood, and when in the tissue they differentiate into macrophages (Rozner et al., 2009). We show that insulin stimulates several cellular pathways that lead to monocyte chemotaxis and their differentiation into macrophages. Manipulation of these pathways may lead to the improvement of insulin-induced wound healing.

## RESULTS

### Insulin stimulates THP-1 cell chemotaxis in an insulin-receptor-dependent manner

**Fig. 1.**
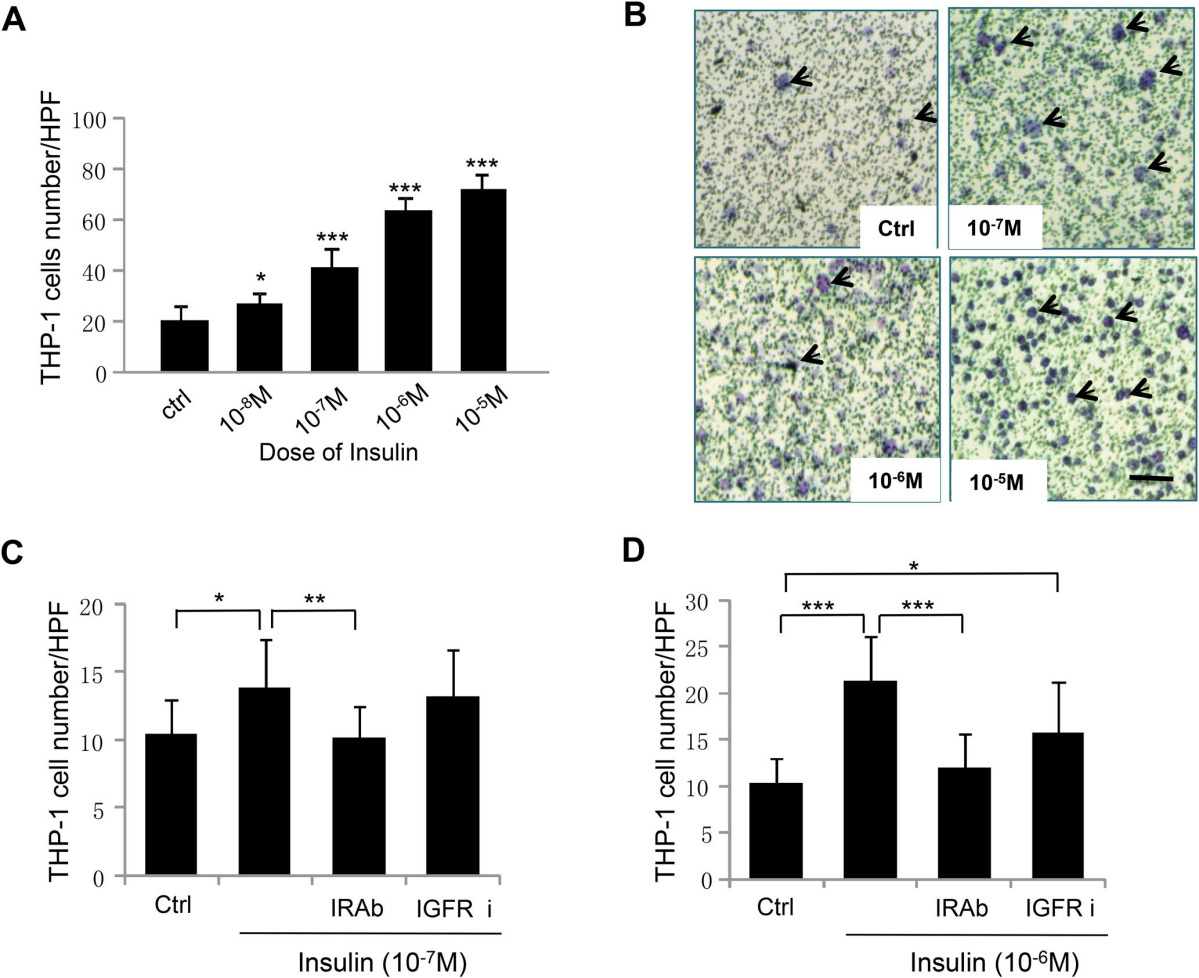
**Insulin stimulates THP-1 cell chemotaxis in an insulin-receptor-dependent manner.** (A) To study the effects of insulin on THP-1 cells chemotaxis, the directional migration towards insulin, THP-1 cells with 1×10^6^ cell number were seeded in the upper chamber of Transwell inserts and then treated with different doses of insulin as indicated for 2 h at 37°C. The chemotactic cells were then stained and counted. *P<0.05, ***P<0.001, *n*=3. Insulin stimulated THP-1 cells chemotaxis in a dose-dependent manner. (B) Representative images of chemotactic THP-1 cells. The green spots are the pores of the transwell membrane and the blue spots are chemotactic THP-1 cells. Arrows point to some of the cells. Scale bar: 50 µm. (C,D) THP-1 cells were pre-treated with 1.5 μg of the neutralizing insulin receptor Ab or 50 nM of IGF-1 receptor inhibitor PPP for 1 h, and then THP-1 cell chemotaxis assay was performed as described above. *n*=3. (C) THP-1cells were treated with 10^−7^ M insulin. *P<0.05, **P<0.01, *n*=3. (D) THP-1cells were treated with 10^−6^ M insulin. *P<0.05, ***P<0.001, *n*=3. For panels A, C and D statistical analysis was performed as described in the Materials and Methods section; data are shown as mean±s.d.

THP-1 cells were seeded in the upper chamber of 8-µm pore transwell filters. Different concentrations of insulin were introduced into the lower chamber and chemotaxis assays performed for three hours. We quantified chemotaxis by counting the number of cells found in the bottom surface of the filters. Increased number of THP-1 cells were found in the bottom surface of the filters when insulin was introduced in the lower chamber. The cell number increased significantly as the concentration of insulin increased (Fig. 1A,B). Because 10^−7^ M insulin showed a highly significant effect on accelerating THP-1 cell migration, we chose this concentration of insulin for all of the subsequent experiments we present here.

Insulin receptor (IR) and insulin-like growth factor (IGF)-1 receptor (IGFR) share high-sequence homology (Garza-Garcia et al., 2007). Our previous work showed that insulin stimulates HaCaT and HMEC-1 cell migration through both IR and IGFR, although with different affinities and with different doses of insulin treatment (Dhall et al., 2015; Liu et al., 2009a). To detect the receptor(s) mediating insulin-induced THP-1 chemotaxis, we performed receptor-inhibition experiments. Blocking IR by pre-treating THP-1 cells with IR neutralizing antibody in the presence of 10^−7^ M insulin treatment significantly inhibited insulin-induced THP-1 cell chemotaxis, while the IGFR inhibitor Picropodophyllin (PPP) showed no effect with the same conditions (Fig. 1C). When cells were treated with 10^−6^ M insulin (Fig. 1D), treatment with PPP partially inhibited THP-1 cells chemotaxis, suggesting that insulin stimulates monocyte chemotaxis in both an IR- and IGFR-mediated manner, with higher doses of insulin functioning through both IR and IGFR.

### PI3K-Akt, SPAK/JNK and p38 mitogen-activated protein kinase (MAPK) signals are involved in insulin-induced THP-1 cell chemotaxis

**Fig. 2.**
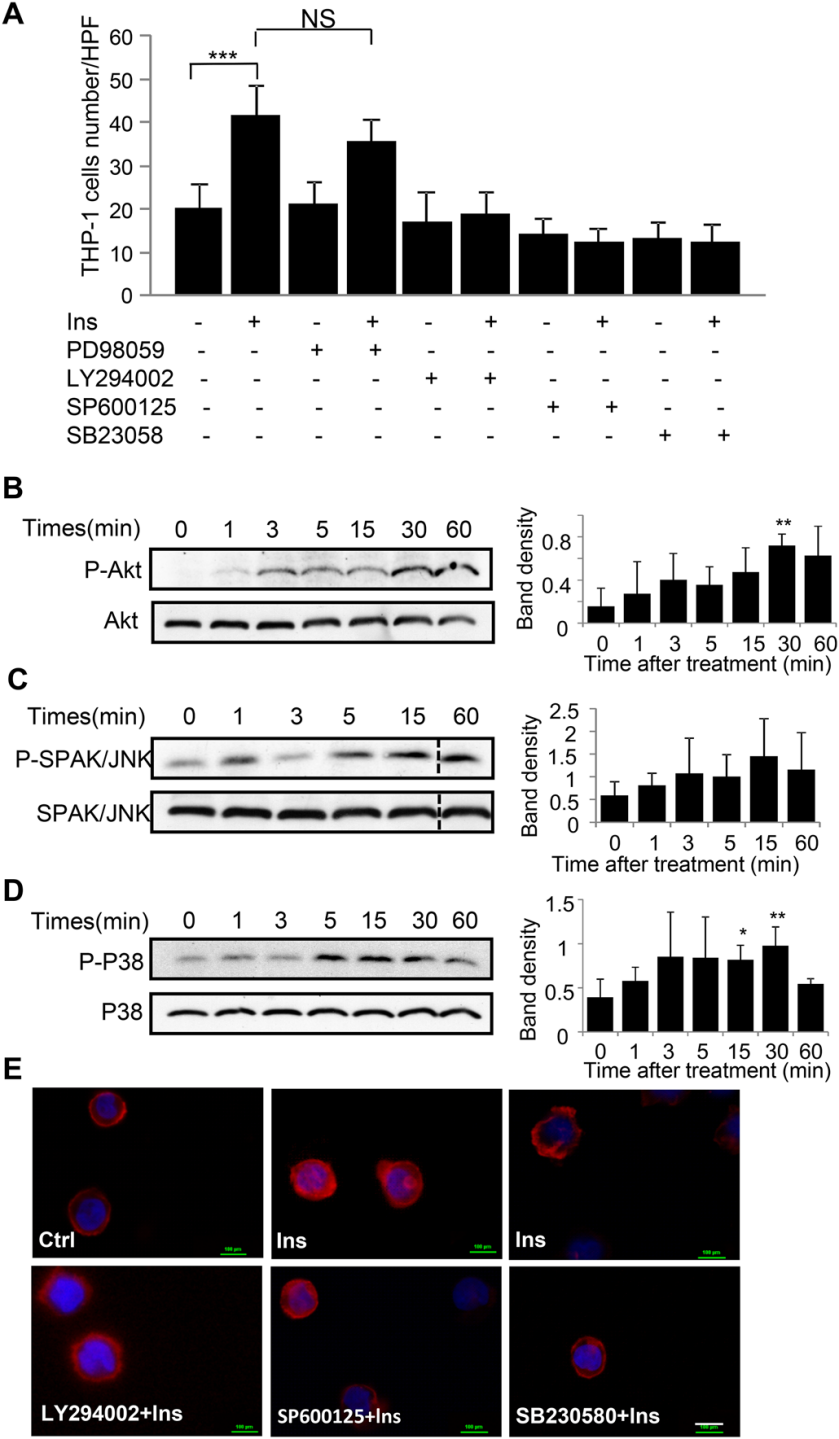
**PI3K-Akt, SPAK/JNK and p38 mitogen-activated protein kinase (MAPK) signals are involved in insulin induced THP-1 cell chemotaxis.** (A) To detect the signals involved in insulin induced THP-1 cell chemotaxis, THP-1 cells were pre-treated with 20 μM ERK inhibitor PD98059, 50 μM PI3 K inhibitor LY294002, 50 μM SPAK/JNK inhibitor SP600125 and 25 μM P38 inhibitor SB203580 for 1 h followed with 10^−7^ M insulin treatment. Chemotaxis assay was performed as described in Fig. 1. ***P<0.001, *n*=3. Statistical analysis was performed as described in the Materials and Methods section; data are shown as mean±s.d. (B-D) THP-1 cells were either left untreated or treated with 10^−7^ M insulin for the indicated time points, cells were then centrifuged and collected and followed by western blot analysis of cell pellet using the antibodies that specifically recognized phosphorylated Akt at Ser 473, phosphorylated SPAK/JNK and phosphorylated p38. These blots were reprobed with corresponding non-phosphorylated antibodies to ensure equal loading. Bar graphs present quantitation of phosphorylated Akt, SPAK/JNK and p38 signal intensity; data are presented as mean±s.d. (*n*= 3), *P<0.05, **P<0.01 compared to basal level. Insulin increased Akt, SPAK/JNK and P38 phosphorylation over time, with (B) peak Akt phosphorylation seen at 30 min; (C) peak SPAK/JNK phosphorylation seen at 15 min; (D) peak P38 phosphorylation seen at 30 min. (E) THP-1cells were pre-treated with 50 μM PI3K inhibitor LY294002, 50 μM SPAK/JNK inhibitor SP600125 and 25 μM P38 inhibitor SB203580 for 1 h followed with 10^−7^ M insulin treatment. Cell migration was then monitored by stained F-actin of cells with Rhodamine phalloidin. Experiments B-E are representative of three different experiments. Scale bar: 10 µm.

To determine the signaling pathways involved in insulin-induced THP-1 cell chemotaxis, we pre-treated the cells with specific pathway inhibitors and then performed transwell cell chemotaxis assays. The inhibitors were chosen from those that are commonly reported affecting monocyte/macrophage motility (Noma et al., 2009). The dosages of the inhibitors, which are sufficient to block the activation of specific target signaling pathways with no obvious cytotoxicity in THP-1 cells, were chosen from previously published work done using THP-1 cells (Xian et al., 2016; Quan et al., 2015; Leyva-Illades et al., 2012). PI3K-Akt inhibitor LY294002, SPAK/JNK inhibitor SP600125 and p38 inhibitor SB230580 pre-treatment completely inhibited insulin-induced THP-1 cell chemotaxis, suggesting that PI3K-Akt, SPAK/JNK, and p38 are involved in insulin-induced monocyte chemotaxis. However, pre-treatment with the MAPK/ERK inhibitor, PD98058, did not affect insulin-induced THP-1 cell chemotaxis, suggesting that this kinase is not involved in insulin-induced monocyte chemotaxis (Fig. 2A). Insulin-induced PI3K-Akt, SPAK/JNK, and p38 signal activation was detected by western blot analysis (Fig. 2B-D). Slight increase in phosphorylated Akt was found after treatment with insulin for 3 min, reached a peak at 30 min of treatment, and lasted for at least 60 min. Phosphorylation of SPAK/JNK lasted for more than 60 min (Fig. 2C), whereas phosphorylation of p38 began to decrease by 60 min of insulin treatment (Fig. 2D). The involvement of PI3K-Akt, SPAK/JNK, and p38 signal in insulin-induced monocyte chemotaxis were confirmed by F-actin staining (Fig. 2E). THP-1 cells became asymmetric after insulin stimulation due to the formation of lamellipodia and filopodia cell protrusions. However, most cells showed round and sharp symmetry when cells were pretreated with PI3K-Akt, SPAK/JNK and p38 inhibitors, and the asymmetric cell ratio in control group, insulin-treated, PI3K-Akt, SPAK/JNK and p38 inhibitors pre-treated cells were 25.7%, 40%, 18.18%, 20% and 25%, respectively, suggesting that these signals were involved in insulin-induced monocyte chemotaxis.

### Insulin-induced Rac1 activation is regulated by PI3K-Akt and SPAK/JNK signal but not p38 in THP-1 cells

**Fig. 3.**
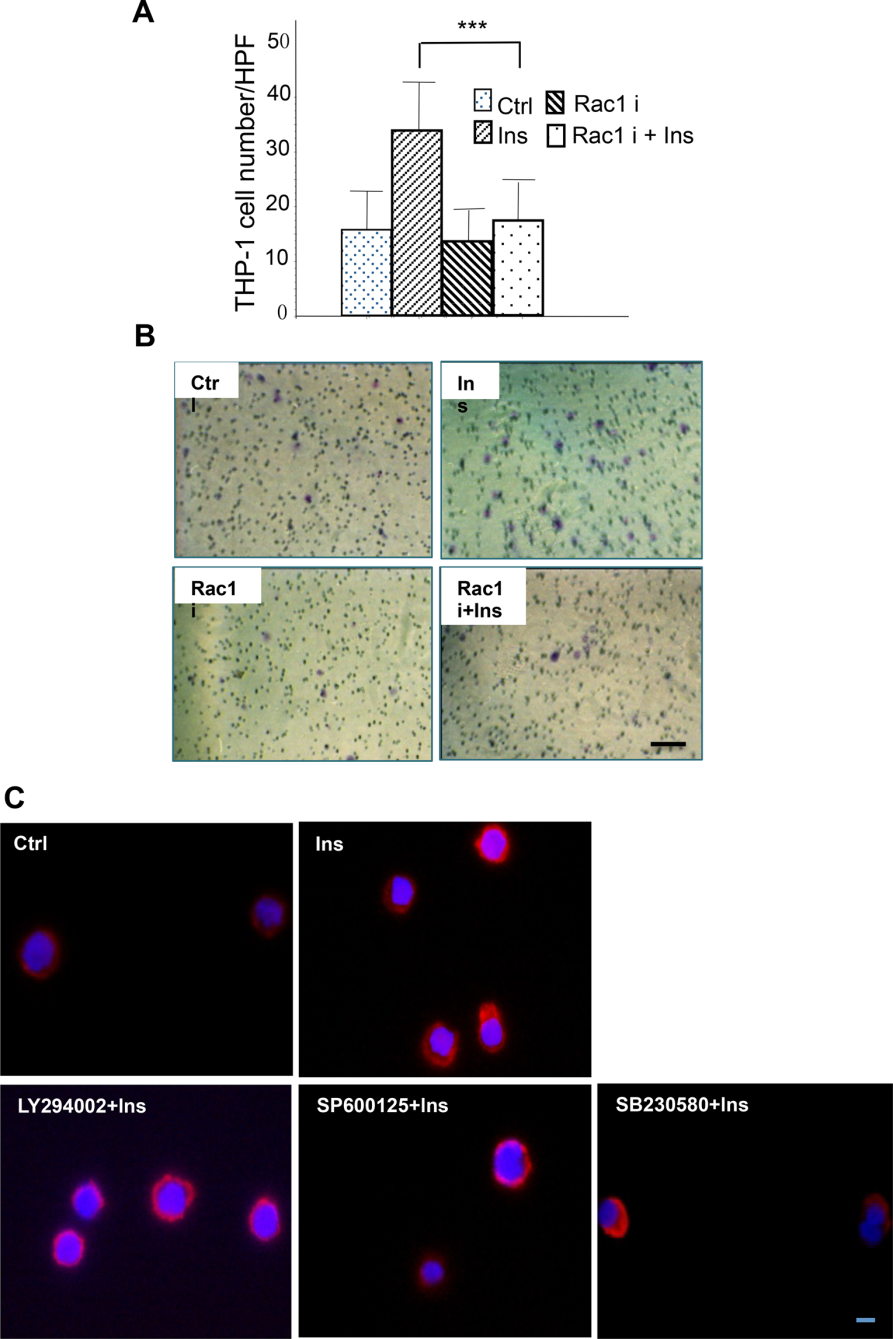
**Insulin-induced Rac1 activation is regulated by PI3K-Akt and SPAK/JNK signal but not p38.** (A,B) THP-1 cells were pre-treated with 50 μM Rac1 inhibitor NSC23766 for 30 min, chemotaxis assay was then performed with or without 10^−7^ M insulin as described in Fig. 1. Insulin-induced chemotaxis was inhibited by Rac1 inhibitor. ***P<0.001, *n*=3. Statistical analysis was performed as described in the Materials and Methods section; data are shown as mean±s.d. (B) Represented pictures of chemotaxis of THP-1 Cells. (C) Cells were pre-treated with 50 μM PI3K inhibitor LY294002, 50 μM SPAK/JNK inhibitor SP600125 and 10 μM P38 inhibitor SB203580 for 1 h, followed by treatment with 10^−7^ M insulin for 15 min. Cells were then collected and fixed, and were stained using Rac1–TRITC antibody. Rac1 distribution was visualized by immunofluorescence microscopy. Insulin-induced Rac1 distributed to the leading edge of migrating cells, which could be inhibited by PI3k and SPAK/JNK inhibitors but not P38 inhibitors. C is representative of three different experiments. Scale bar: 10 µm.

Small GTPase, Rac1, has been found to be closely associated with cell motility. Pre-treatment with the Rac1-specific inhibitor, NSC23766, abolished insulin-induced monocyte chemotaxis which suggests that Rac1 is involved in insulin-induced monocyte chemotaxis (Fig. 3A,B). These results were confirmed using Rac1 immunostaining. Insulin treatment caused increased Rac1 presence at the leading edge of the monocytes (Ins) (leading edge Rac1-enriched cell ratio is 29.03%). The same Rac1 distribution was found in p38 MAPK inhibitor pre-treated cells (SB230580) (Rac1-enriched cell ratio is 34.48%). However, even distribution of Rac1 was observed when cells were pre-treated with PI3K-Akt (LY294002) and SPAK/JNK inhibitors (SP600125), suggesting that insulin-induced Rac1 activation is mediated by both PI3K-Akt and SPAK/JNK signal (Fig. 3C).

### Rac1 is involved in insulin-induced monocyte chemotaxis to wound tissue

**Fig. 4.**
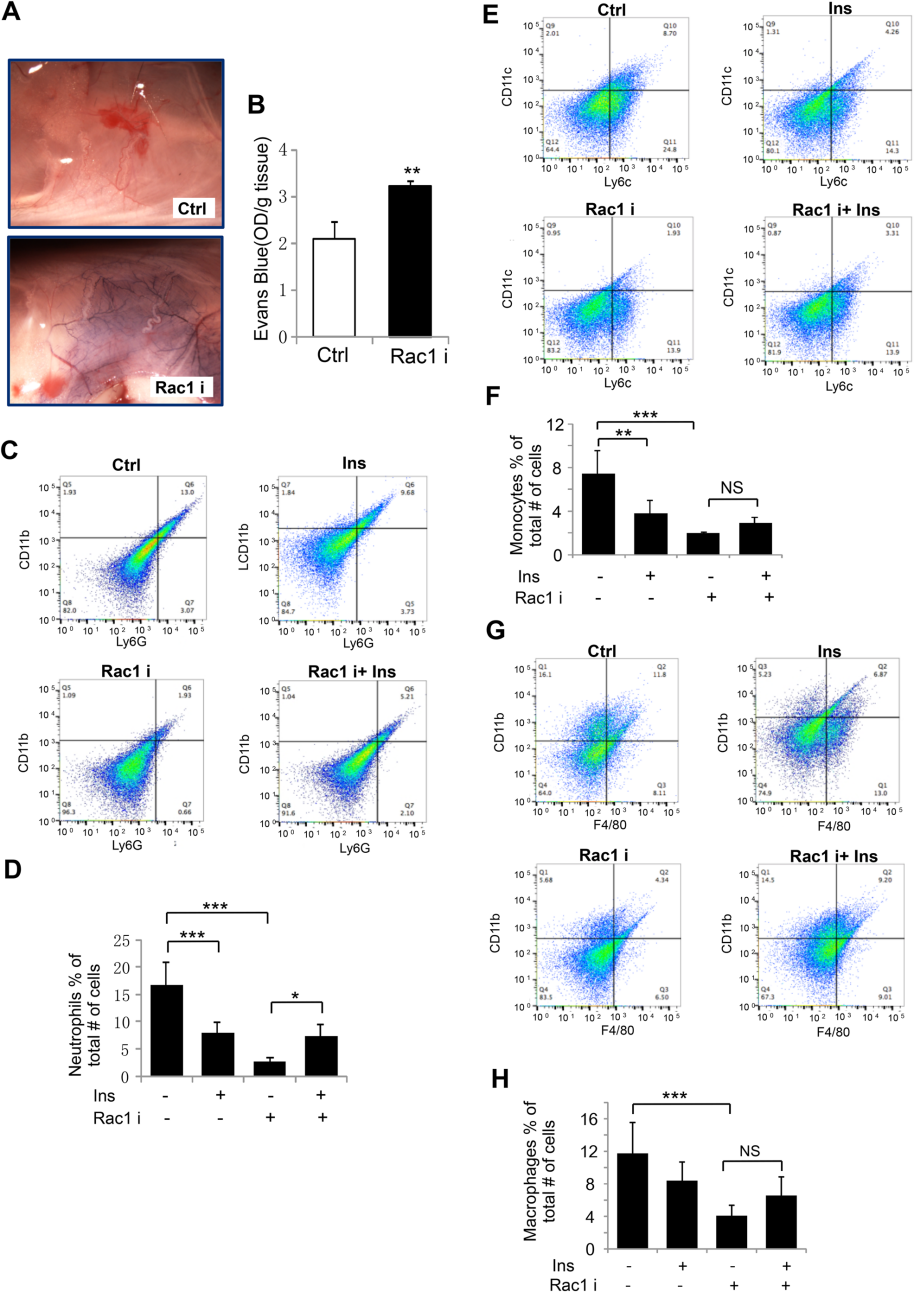
**Rac1 is involved in insulin-induced monocyte/macrophage chemotaxis to wound area.** (A) 7-mm excision wounds in C57BL/6 mice were either treated with 20 μl saline or 50 μg/20 μl Rac1 inhibitor NSC23766 solution for 3 days, consecutively. 100 μl of 2% Evans Blue dye in sterile normal saline was then introduced via tail vein injection. Post 30 min, a 10-mm punch biopsy along with surrounding normal tissue was excised and photographed to visualize the Evans Blue color where microvessels leaked (*n*=6). (B) To quantify Evans Blue staining, the excised tissue was incubated for 24 h with 400 μl of formamide to extract the dye which was quantified using a spectrophotometer at 600 nm. Absorbance was normalized to tissue weight. (C-H) Excisional wounds on C57BL/6 mice were either treated daily with 20 μl saline or 0.03 U insulin alone. Separate wounds were pre-treated with 50 μg/20 μl Rac1 inhibitor NSC23766 for 30 min prior to injecting 0.03 U insulin. Skin tissues were collected 3 days post wounding and isolated cells underwent antibody conjugated differential flow cytometry analysis. Inflammatory cells were stained with anti ly6G, ly6C, CD11b, CD11c and F4/80 antibody. Three time FACS analysis were performed, (*n*=3 for each assay). (C) Representative FACS-plot for ly6G/CD11b positive neutrophils. (D) Proportion of wound neutrophils. (E) Representative FACS-plot for ly6C/CD11c positive monocytes. (F) Proportion of wound monocytes. (G) Representative FAC S-plot for F4/80/CD11b positive macrophages. (H) Proportion of wound macrophages. Analysis of the results was performed using FlowJo software that contains the necessary tools for generation graphs and statistical reports. For panels B, D, F and G, statistical analysis was performed as described in the Materials and Methods section; data are shown as mean±s.d. *P<0.05, *P<0.001.

To confirm that Rac1 mediates insulin-induced wound monocyte/macrophage chemotaxis, we performed Rac1 inhibition experiment *in vivo*. Because it has been suggested that activation of small GTPase Rac1 stabilizes the endothelial barrier (Aslam et al., 2011), we first examined the effect of Rac1 inhibition on wound blood vessel permeability. Rac1 inhibition caused an increase in blood vessel permeability shown by significantly elevated level of Evans Blue dye at the site of injection of Rac1 inhibitor under the skin (Fig. 4A,B).

We then examined the inflammatory cell infiltration into the wound tissue using FACS analysis. The cell populations were defined with markers for neutrophils as having (CD11b+, Ly6G+), monocytes (CD11c+, Ly6C+), and macrophages (CD11b+, F4/80+). The number of neutrophils, monocytes, and macrophages were quantified in absolute number and then the % was calculated based on the total number of cells collected and read during FACS analysis. Decreased neutrophil (CD11b+, Ly6G+) infiltration was found in insulin-treated wounds at day 3 post-wounding. Treatment with Rac1 inhibitor alone further decreased the number of neutrophils in the wound tissue, suggesting that the inhibition of neutrophil chemotaxis occurred due to the inhibition of Rac1. However, the decrease in neutrophil infiltration observed with Rac1 inhibitor treatment was reversed by insulin treatment. This suggested that signaling pathways other than Rac1 are involved in insulin-stimulated neutrophil chemotaxis (Fig. 4C,D).

We then determined the behavior of monocyte infiltration. A significant decrease in monocyte (CD11c+, Ly6C+) infiltration at day 3 post-wounding was observed in insulin-treated wounds as compared to saline-treated control wounds. Rac1 inhibitor significantly inhibited monocyte chemotaxis. However, insulin treatment did not significantly increase monocyte chemotaxis, suggesting Rac1 is the primary signaling molecular-mediating insulin-induced monocyte infiltration to wound site (Fig. 4E,F). Furthermore, there was no significant difference in macrophage number between the control and insulin-treated wound, albeit a slight decrease in macrophages was seen in insulin-treated wounds (Fig. 4G,H). Moreover, insulin couldn't rescue Rac1 inhibitor-induced reduction in wound macrophages, suggesting that Rac1 is also the main signaling molecule mediating insulin-induced monocyte infiltration and then differentiation into the wound tissue (Fig. 4G,H).

These results show that Rac1 inhibition significantly inhibits neutrophil infiltration and that this inhibition can be reversed by insulin treatment, suggesting that other signaling molecules are involved in insulin-induced neutrophil chemotaxis. Contrary, insulin was not able to significantly improve monocyte infiltration in the presence of Rac1 inhibitor, suggesting that Rac1 is the principal regulator of insulin-induced monocyte chemotaxis.

### Rac1 inhibition during the early stages of wound healing inhibited insulin-induced wound healing

**Fig. 5.**
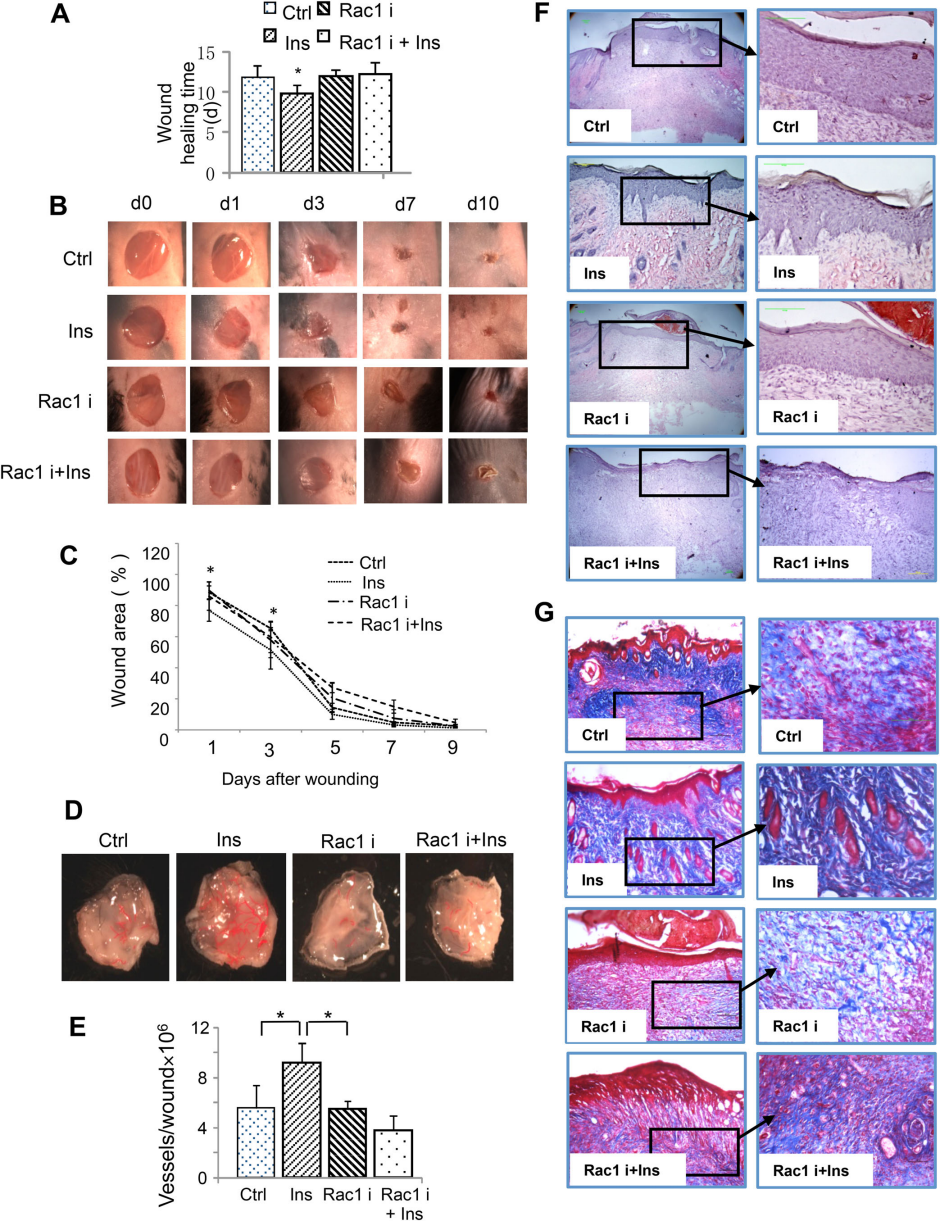
**Rac1 inhibition at early stage of wound healing greatly obstructed insulin-induced wound healing.** (A) Four 7-mm diameter excision wounds were made on the back of C57/BJ mice. Two wounds were then treated with 50 μg/20 μl saline Rac1 inhibitor NSC23766 for 30 min followed with/without 0.03 U insulin treatment, another two wounds were treated with either 20 μl saline or 0.03 U insulin. Treatment was applied every day for five consecutive days after wounding. Skin tissue was collected after complete healing was achieved. Healing time was recorded. *n*=6 for each treatment. (B) Representative images of wounds which were treated with vehicle (30 μl saline solution), 0.03 u insulin/30 µl saline solution, NSC23766 50 μg/30 μl saline solution or NSC23766 50 μg+0.03 U insulin/30 μl saline solution. (C) Wound area was quantified every two days and expressed as the percentage of the original wound area. Statistics are shown as comparisons between the treatment and control. *n*=6 for each treatment. (D) Representative images of skin treated with insulin, Rac inhibitor alone and insulin with Rac inhibitor. The treatments were applied for 4 consecutive days under the skin using an insulin syringe. The skin was then excised and the microvessels were highlighted with red lines to facilitate analysis. (E) Image J software was used to quantitatively analyze microvessel number and thickness. *n*=4. (F) Representative hematoxylin and eosin-stained sections showing increased number of epidermal reticular ridges and dermal papilla in insulin-treated healed wounds suggested a better healing achieved by insulin. Insulin-improved healing was inhibited by Rac1 inhibitor NSC 23766 pre-treatment. *n*=6. (G) Representative Masson's trichrome staining sections showing increased and more mature collagen fibers were found in insulin-treated healed wounds. Insulin-improved healing was inhibited by Rac1 inhibitor NSC 23766 pre-treatment. *n*=6. For panels A, C and E, statistical analysis was performed as described in the Materials and Methods section; data are shown as mean±s.d. *P<0.05.

Rac1 inhibitor was applied during the first five days after wounding. Insulin-induced accelerated wound healing was inhibited by Rac1 inhibition, as shown by longer healing time (Fig. 5A), and delayed wound closure (Fig. 5B,C), as compared to insulin treatment alone. An increase in blood vessels and vessel networking was observed in insulin-treated skin tissue. NSC23766 (inhibitor for Rac1) treatment alone caused a decrease in blood vessel development, whereas insulin application was not capable of rescuing NSC23766-induced inhibition of blood vessel development (Fig. 5D). Quantitative analysis of both blood vessel number and vessel diameter confirmed these findings (Fig. 5E). The quality of the healing was assessed by histological evaluation using H&E staining (Fig. 5F). A thick, continuous and complete epidermal layer in both control and Rac1-inhibitor-treated wounds was observed suggesting that complete healing was achieved. Similar to our previously reported results (Liu et al., 2009b), retes were seen in the epithelium and the dermal epidermal interaction was well established in insulin-treated wounds. This suggested that an improvement in differentiation and maturation of the epidermis-dermal junction was achieved when wounds were treated with insulin. However, delayed and poor healing, with a barely covered epidermis, was found in Rac1-inhibitor-treated wounds even in the presence of insulin (Fig. 5F). Masson trichrome staining was used to visualize the quality and quantity of collagen. Newly formed thin and light blue collagen fibers were found in control wounds, whereas in insulin-treated wounds, thick and well-organized dark blue collagen fibers were found, suggesting a more mature collagen formation upon stimulation of insulin. However, in the Rac1-inhibitor-treated wounds, fewer collagen fibers were found and they had diminished maturity. This was also seen when insulin treatment was done in the presence of the Rac1 inhibitor (Fig. 5G).

Taken together, Rac1 inhibition during the early stages after wounding significantly affected insulin-induced wound healing. Inflammatory cell infiltration, blood vessel formation, and epidermal-dermal maturation and differentiation were all inhibited by Rac1 inhibitor.

## DISCUSSION

**Fig. 6.**
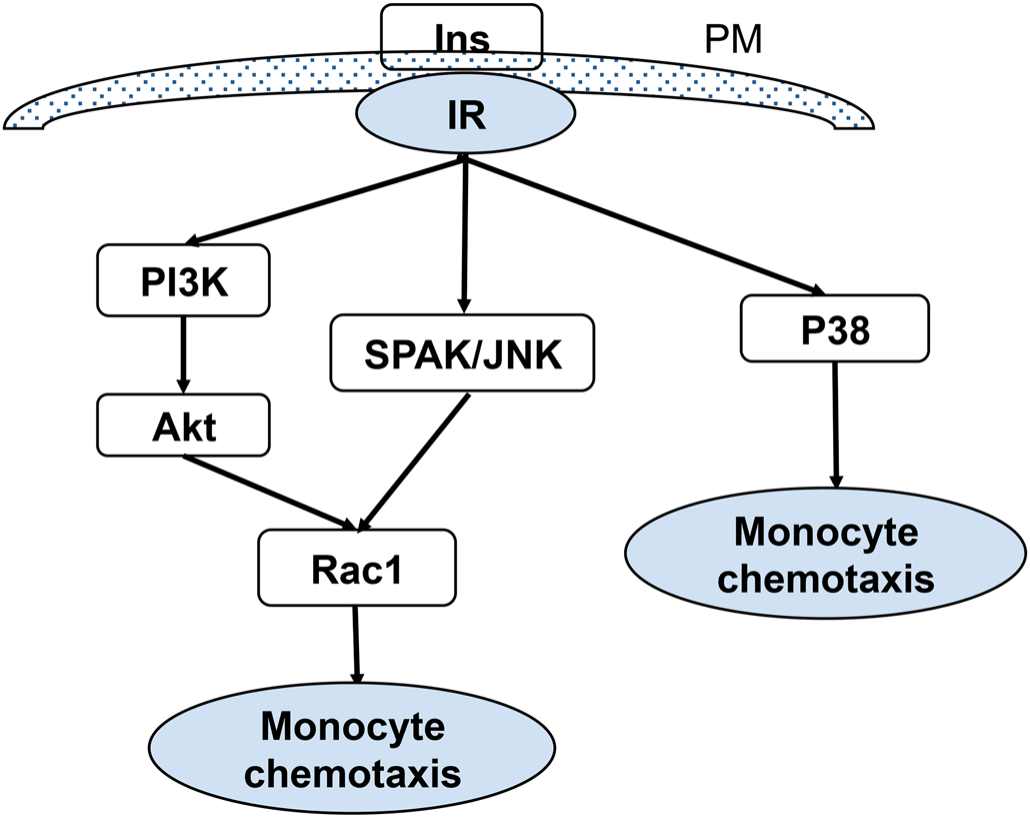
**Schematic representation of multiple signaling pathways activated by insulin leading to monocyte/macrophage chemotaxis.** Insulin stimulates THP-1 cell chemotaxis in an insulin-receptor-dependent manner. PI3K-Akt, SPAK/JNK, and p38 MAPK are signal pathways that are involved in insulin-induced THP-1 cell chemotaxis. Rac1 is an important molecule in regulating cell motility. Both PI3K-Akt and SPAK/JNK signaling are involved in Rac1 activation.

Monocyte/macrophages are vital regulatory cells during wound healing. Our previous work showed that insulin improves wound healing by stimulating monocyte/macrophage chemotaxis. Here, we used a THP-1 cell, a monocyte/macrophage cell line, and a variety of approaches to elucidate the signaling pathways involved in insulin-induced THP-1 cell chemotaxis. We found that insulin stimulates THP-1 cell chemotaxis in a dose-dependent and insulin-receptor-dependent manner. PI3K-Akt, SPAK/JNK, and p38 MAPK signaling pathways are involved in the insulin-induced THP-1 cell chemotaxis. Furthermore, both PI3K-Akt and SPAK/JNK signaling are involved in Rac1 activation, an important molecule in regulating cell motility (Fig. 6). Topical inhibition of Rac1 at an early stage during the healing process caused delayed and impaired healing. We also found that interaction of insulin with its receptor activates p38, directly leading to monocyte/macrophage chemotaxis (Fig. 6).

Monocytes are a subset of leukocytes produced in the bone marrow. They circulate in the blood and, when needed, migrate to injured tissues upon the stimulation by chemokines and other inflammatory mediators. After recruitment to the wound bed, monocytes differentiate into macrophages and orchestrate the wound healing process. By means of phagocytosis and the production of inflammatory cytokines, macrophages first perform pro-inflammatory functions, and then they accelerate resolution of inflammation. In addition, macrophages produce cytokines and growth factors, such as IL-1, IL-6, TNF-α, TGFα, PDGF, VEGF and EGF (Mahdavian Delavary et al., 2011; Novak and Koh, 2013), that stimulate proliferation, migration or differentiation of fibroblasts, keratinocytes and endothelial cells, and ultimately stimulate ECM deposition, re-epithelialization and neovascularization. In several genetically modified mice models, macrophage depletion resulted in defects in re-epithelization, granulation tissue formation, and angiogenesis resulting in impaired healing (Mirza et al., 2009; Goren et al., 2009). Moreover, the crosstalk between macrophage and wound repair cells, i.e. keratinocytes, endothelial cells and fibroblasts, play an important role during the healing process. Our previous work showed that insulin accelerates re-epithelialization and angiogenesis by stimulating keratinocyte and endothelial cells migration (Liu et al., 2009a,b). Insulin stimulates keratinocyte LN332 production and induces ‘maturation’ of the healing tissue characterized by well-differentiated epidermal and epidermal–dermal junction, as well as increased blood vessels networking, which is possibly related to insulin-regulated monocyte/macrophages as well as keratinocytes and endothelial cell function.

Chemokines are major molecules that attract and stimulate monocyte directional migration to the wound site. Macrophage inflammatory protein-1α/CC chemokine ligand 3 (MIP-1α/CCL3), MIP1-β (CCL4), stromal cell-derived factor -1(SDF-1/CXCL12) and RANTES (CCL5) are all potent chemoattractants of monocyte/macrophage chemotaxis. Through binding and activation of chemokine receptors, chemokines activate multiple down-stream signaling molecules and signaling pathways that result in monocyte/macrophage chemotaxis. Phospholipase C (PLC) (Kang et al., 2014), protein kinase C (PKC) δ (Kim et al., 2005), phosphoinositide 3-kinase (PI3K)-Akt, p38 MAPK (Kim et al., 2005; Ruth et al., 2010), and ERK1/2 (Gouwy et al., 2014) (p44/42 MAPK, Malik et al., 2009) have all been reported to be involved in chemokine-activated monocyte/macrophage chemotaxis. Our results suggested that PI3K-Akt, SPAK/JNK, and p38 MAPK signaling pathways are involved in the insulin-induced monocyte chemotaxis. It has been previously reported that insulin treatment for 12 h, in the gelatin-coated membrane system, facilitated human monocytic THP-1 cell chemotaxis via prolonged ERK1/2-dependent induction of MMP-9 (Kappert et al., 2008). These studies cannot be considered as analyzing chemotaxis because the chemotactic gradient will have disappeared after 3 h. These are more like chemokinesis studies. This is probably the reason why we did not find that ERK1/2 was involved in insulin-induced THP-1 cell chemotaxis. The difference in time course and the presence of ECM are most likely the reasons for the discrepancy between the previous studies and the study presented here.

Although we detected an insulin-induced increase in monocyte chemotaxis *in vitro*, we did not observe an upsurge in monocytes/macrophages infiltration in insulin-treated wound areas at day 3 after wounding, the day we performed FACS analysis. Instead, we found significantly decreased neutrophils and monocytes, suggesting controlled inflammatory response after insulin treatment. Decreased neutrophil infiltration could possibly be due to the low level of MIP-2 production by insulin treatment, and possibly other chemokines, as we found in our previous study (Liu et al., 2009b). We also recognize that in addition to chemokines, other factors also affect wound inflammatory cells. The anti-apoptotic effect of insulin has been well described (Farrelly et al., 1999); and our recent work found that insulin decreased production of reactive oxygen species in the wound tissue (Dhall et al., 2015). Therefore, insulin treatment that resulted in a ‘healthier’ wound bed could lead to faster resolution of inflammation at day 3 post wounding. Further, faster initiation and resolution of the macrophages, induced by insulin, was previously observed in our kinetic study (Chen et al., 2012b). However, due to the complexity of FACS analysis, specifically cell extraction and multiple marker staining processes, we only performed and analyzed a single time point in this study.

To confirm the role of Rac1 in insulin-induced monocyte/macrophage chemotaxis, we used Rac1 inhibitor to abolish the effect of Rac1 and then detected wound monocyte/macrophage infiltration when wounds were treated with insulin. Considering the essential role of GTPases Rac1, cdc42, and Rap in barrier maintenance and stabilization (Curry and Adamson, 2013; Spindler et al., 2010), we first detected the effect of Rac1 inhibition on blood vessel permeability using Evans Blue assay. Evans Blue is a high affinitive dye for serum albumin. Under normal conditions, injected Evans Blue is bound to serum albumin and leaves normal tissue unstained because albumin cannot cross the vessel barrier. In cases of compromised vessel barrier, albumin-bound Evans Blue leaves the blood vessels and enters the surrounding tissue. Measuring the quality of Evans Blue in extra-vessel tissue thus becomes a manner of estimating blood vessel leakage. After topical application of NSC 23766, a specific inhibitor of Rac1, we found clear vessel leakage in the wound areas at day 3 post-wounding. This increased blood vessel permeability caused by injury which otherwise should have been completely recovered. It is highly possible that Rac1 inhibition increases vessel permeability that allows the inflammatory cells to move easily through the blood vessels and migrate to wound sites. The increased vessel permeability makes our *in vivo* cell chemotaxis more similar to ‘*in vitro* chemotaxis’, where cell chemotaxis is possibly determined by wound chemoattractants. Rac1 inhibition decreased the number of wound neutrophils at day 3 post-wounding, suggesting that Rac1 is also involved in neutrophil chemotaxis; however, significant increases in neutrophil in the presence of insulin strongly suggested alternative signaling was involved in insulin-induced neutrophil chemotaxis. Rac1 inhibition significantly decreased wound monocyte/macrophage infiltration, confirming the role of Rac1 in the chemotaxis of these inflammatory cells. Our previous *in vitro* study on insulin-induced THP-1 cell chemotaxis proposed two pathways of insulin signaling on monocyte/macrophage migration: (i) a general effect on cell motility, and (ii) a specific chemotactic effect on monocyte chemotaxis (Chen et al., 2012b). Hence, we propose that the slight increase in monocyte/macrophage infiltration in the wound area might be due to the general effect of insulin on cell motility. However, the increase in monocyte/macrophage infiltration is not significant, as it is in neutrophil, in the presence of insulin, suggesting that Rac1 is the main signaling molecule involved in insulin-induced monocyte/macrophage chemotaxis. Furthermore, *in vivo*, we found a delayed rate of closure and impaired healing quality in Rac1 inhibition wounds, strongly suggesting that Rac1 is a key molecule for the effects of insulin that improve the quality of healing. However, the impaired healing observed with Rac1 inhibition, that was even more noticeable when insulin was present, cannot be explained by a deficient inflammatory response since inflammatory cell infiltration was very similar between insulin-treated and Rac1 inhibitor combined with insulin-treated wounds. It is known that Rac1 is also involved in insulin induced endothelial cell and keratinocyte migration (Liu et al., 2009a,b). Therefore, it is possible that impaired healing was due to the inhibition of endothelial and keratinocyte cell migration and other cell functions. It has been reported that deletion of Rac1 results in embryonic lethality in midgestation [embryonic day (E) 9.5], with multiple vascular defects (Nohata et al., 2016). These investigators also found that Rac1 is spatially involved in endothelial cell migration, invasion, and radial sprouting activities in a 3D collagen matrix *in vitro* model (Nohata et al., 2016). Insulin stimulation of integrin α3 and LN332 in keratinocytes is involved in epidermal-dermal junction construction (Liu et al., 2009b). The poor healing quality caused by Rac1 inhibition provides the possibility that Rac1 signaling is involved in the assembly of epidermal-dermal junctions and formation of basement membrane. All these results suggest a broad effect of Rac1 on a variety of cell types during the healing process.

Taken together, these studies show that insulin stimulates THP-1 cell chemotaxis in a dose- and insulin receptor-dependent manner. Also, PI3K-Akt, SPAK/JNK, and p38 MAPK signal pathways were involved in insulin-induced THP-1 cell chemotaxis. Furthermore, both PI3K-Akt and SPAK/JNK signals are involved in Rac1 activation, which is an important molecule in regulating cell motility whereas p38 does not use Rac1 for its effects (Fig. 6).

## MATERIALS AND METHODS

### Reagents

Bovine thrombin was purchased from Fisher Bioreagents (Fair lawn, NJ), recombinant human insulin from Sigma-Aldrich (St. Louis, MO) and recombinant human insulin (humulin) isophane suspension from Eli Lilly and Company (Indianapolis, IN). Transwell systems were purchased from BD Biosciences (Franklin Lakes, NJ), rhodamine-phalloidin from Invitrogen (Carlsbad, CA). IGF-1R Inhibitor Picropodophyllin (PPP) from Santa Cruz Biotechnology (Dallas, TX; cat #477-47-4), Rac1 inhibitor NSC 23766 from Cayman Chemical (Ann Arbor, Mi; cat #23766), ERK inhibitor PD98059 (cat #9900), PI3K inhibitor LY294002 (cat #9901), P38 inhibitor SB23058 (cat #8158) and SPAK/JNK inhibitor SP600125 (cat # 8177) from Cell Signaling Technology (Danvers, MA). Percoll was supplied by Sigma-Aldrich. The following antibodies were obtained from various suppliers: anti-insulin receptor (cat #29B4), phospho-Akt and Akt (cat #9272), phospho-SPAK/JNK and SPAK/JNK (cat #9255), phospho-P38 (cat #9216) and P38 (cat #9212) (Cell Signaling Technology, Danvers, MA), Rac1-TRITC (BD Biosciences, Franklin Lakes, NJ; cat #610651). All anti-mouse antibodies for FACS and OneComp eBeads were from eBioscience (San Diego, CA): CD16/CD32, CD11c PE-eFluor^®^610, IgG Isoytpe control PE-eFluor^®^610, Ly-6C APC, IgG1K Isoytpe control APC, Ly-6G(Gr-1) PerCP-Cyanine5.5, IgG2b K isotype PerCP-Cyanine5.5, F4/80 FITC, IgG1K isoytpe control FITC, CD11b PE-Cyanine7, IgG1K Isoytpe control PE-Cyanine7, CD11c Alexa Fluor^®^532, IgG Isotype control Alexa Fluor^®^532.

### *In vivo* wound model

C57BL/6J mice were purchased from The Jackson Laboratory (USA), and housed at the University of California, Riverside (UCR) vivarium. All experimental protocols were approved by the UCR Institutional Animal Care and Use Committee. Experiments were performed in 8–12-week-old mice. The mice were anesthetized with a single intraperitoneal injection of ketamine (80 mg/kg body weight)/xylazine (16 mg/kg body weight). Full-thickness 7-mm punch wounds (excision of the skin and the underlying panniculus carnosus) were made on the back of the mice. The wounds were then treated as indicated for the various experiments. A transparent dressing (Bioclusive, Johnson & Johnson Medical Limited, USA) was used to cover the wound area for the first three days after wounding to ensure better absorbing of the treatment solution. Samples were collected at day 3 after wounding for FACS analysis, and also collected on the day of complete healing for histological analysis. The mice were then euthanized using CO_2_. The mice were excluded if any signs of wound infection, including wound redness, swelling and cloudy exudation were observed. For FACS analysis, wound tissues, along with adjacent normal skin were harvested. For histological observation, full-thickness punch wounds or healed wounds were collected (*n*=6). 10-µm sections were mounted on gelatin-coated microscope slides and were stained with hematoxylin and eosin (H&E) and Masson's Trichrome according to manufacturer's instruction. Blood vessels were highlighted using ImageJ software (NIH, Bethesda, MD) as described before (Chen et al., 2012b).

### Cell culture

Human monocytic THP-1 cells (American Type Culture Collection, Manassas, VA; lot #58636802) were cultured in RPMI-1640 medium (Mediatech Inc., Manassas, VA) with 4.5 g/l glucose, 10 mM HEPES, 1 mM sodium pyruvate, and 50 mM β-ME, supplemented with 10% FBS (Sigma, St. Louis, MO), 10 units/ml penicillin, and 10 μg/ml streptomycin sulfate (GIBCO, Invitrogen Corporation) in a 5% CO_2_ atmosphere at 37°C. The cells were certified by ATCC on November 9th 2009. Certificate can be provided upon request.

### Immunoblotting

Cells were treated as indicated, and collected by centrifugation for 3 min at 3000 ***g*** with one time wash using ice-cold 1× PBS. Cells were lysed on ice with lysis buffer containing 0.5% Triton X100, 0.5% Nonidet P-40, 10 mM Tris, pH 7.5, 2.5 mM KCl, 150 mM NaCl, 30 mM b-glycerophosphate, 50 mM NaF, 1 mM Na3VO4, 0.1% SDS and additional protease and phosphatase inhibitor cocktails (Sigma). Protein concentrations were measured using the DC protein assay kit (Bio-Rad). Equal amounts of protein in the cell extracts were mixed with sample buffer, boiled, and analyzed using 10% acrylamide SDS-PAGE. Immunoblotting was performed with the indicated primary antibodies and the appropriate HRP-conjugated secondary antibodies, followed by incubation with West Dura extended duration substrate (Pierce Biotechnology). Blots were then stripped and re-probed for non-phosphorylation protein or house-keeping proteins to show equal loading.

### *In vitro* chemotaxis assays

Chemotaxis assays were performed in triplicate in 24-well transwell chambers with 8.0 µm pore polycarbonate membrane insert. 1×10^6^ cells were seeded into the upper chamber of transwell, and then treated with different doses of insulin as indicated for 2 h at 37°C. Remaining cells in upper chamber were removed by a cotton swab, and the THP-1 cells on the under side of the filter were stained with 2% Toluidine blue/4% paraformaldehyde for 1 h and the cell numbers counted in five random representative 20× fields under phase microscopy.

### Immunolabeling

Cells were treated as indicated and then fixed in 4% paraformaldehyde for 20 min, rinsed with PBS, incubated in PBS containing 0.1 M glycine for 20 min, and blocked with 3% BSA, 0.1%Triton X-100 in PBS for 30 min. Rac1-TRITC antibody or rhodamine-phalloidin was applied to the cell suspension and incubated for 1 h or 30 min at room temperature, respectively. After washing, the cells were dropped onto glass slides, and mounted in Vectashield containing DAPI (Vector Laboratories, Inc. Burlingame, CA). Immunofluorescence was visualized and imaged using a Leica SP2 laser scanning confocal microscope. For frozen tissues, 8-μm cryosections were washed in 1× PBS to remove the OCT, fixed in 2% paraformaldehyde for 10 min, incubated in 0.1 M glycine in 1× PBS, followed by the primary and secondary antibodies using the same procedure as indicated above.

### Fluorescence activated cell sorting (FACS) analysis

Wounds, along with 5-mm width surrounding tissue, were collected at given time points. Wound tissues were then cut into small pieces with scissors and combined with 100 ml of collagenase/dispase (1 mg/ml), incubated for 45 min at 37°C. The cell suspension was passaged through 18 and 20 gauge needles and then a 70-µm cell strainer (Falcon, BD). Cells were washed with RPMI. The Percoll density gradient method was then used to separate neutrophil and monocyte/macrophage from the cell suspension. Collected cells were washed with RPMI and incubated in 10% purified CD16/CD32 in FACS buffer (10 μl) for 5 min to block non-specific binding to antibodies. Cells were then collected and re-suspended in 100 µl FACS buffer with 1 µl of each antibody and incubated on ice for 30 min followed by FACS analysis using FACS Aria (BD Biosciences). The data were analyzed with the FlowJo software (www.flowjo.com) which contains sophisticated tools that allow generation of graphs and statistical reports.

### Sample size and statistical analysis

To ensure adequate power to detect specific effect, for all cell studies three independent experiments were performed. We considered adequate power if all samples fell within two standard deviations of the mean. For FACS analysis, three independent experiments were performed, each experiment with pooled skin samples from three mice to obtain sufficient number of cells for FACS analysis. For the *in vivo* experiments, making the conservative assumption of a standard deviation of 0.75, we determined that to obtain a power of 0.9 we need six mice per set of experiments plus a comparable number of controls, hence, at least six animals were used. Animals were excluded from the study if any sign of sickness or changes in behavior were observed. Animals were randomly chosen from the colony for experimental and control groups. When the *n*=3 was sufficient to give us statistical significance of the data, we showed in the figures a representative image and then the statistical analysis.

Data analysis was performed using GraphPad Instat software (GraphPad Software Inc.). *t*-tests were used to determine the significance of pair-wise differences between means, unpaired *t*-tests for comparison between two groups and one-way ANOVA (Dunnett's post hoc test) was used to determine significance between means of several groups. Data satisfying the assumptions of ANOVA were verified before performing the tests. The *P*-value less than 0.05 were considered significant statistically, and the *P*-value less than 0.01 were considered statistically highly significant. Data are shown as mean±s.d.
